# The Garlic Allelochemical Diallyl Disulfide Affects Tomato Root Growth by Influencing Cell Division, Phytohormone Balance and Expansin Gene Expression

**DOI:** 10.3389/fpls.2016.01199

**Published:** 2016-08-09

**Authors:** Fang Cheng, Zhihui Cheng, Huanwen Meng, Xiangwei Tang

**Affiliations:** Department of Vegetable Science, College of Horticulture, Northwest A&F UniversityYangling, China

**Keywords:** allelopathy, DADS, mitosis, plant hormones, expansin, tomato

## Abstract

Diallyl disulfide (DADS) is a volatile organosulfur compound derived from garlic (*Allium sativum* L.), and it is known as an allelochemical responsible for the strong allelopathic potential of garlic. The anticancer properties of DADS have been studied in experimental animals and various types of cancer cells, but to date, little is known about its mode of action as an allelochemical at the cytological level. The current research presents further studies on the effects of DADS on tomato (*Solanum lycopersicum* L.) seed germination, root growth, mitotic index, and cell size in root meristem, as well as the phytohormone levels and expression profile of auxin biosynthesis genes (*FZYs*), auxin transport genes (*SlPINs*), and expansin genes (*EXPs*) in tomato root. The results showed a biphasic, dose-dependent effect on tomato seed germination and root growth under different DADS concentrations. Lower concentrations (0.01–0.62 mM) of DADS significantly promoted root growth, whereas higher levels (6.20–20.67 mM) showed inhibitory effects. Cytological observations showed that the cell length of root meristem was increased and that the mitotic activity of meristematic cells in seedling root tips was enhanced at lower concentrations of DADS. In contrast, DADS at higher concentrations inhibited root growth by affecting both the length and division activity of meristematic cells. However, the cell width of the root meristem was not affected. Additionally, DADS increased the IAA and ZR contents of seedling roots in a dose-dependent manner. The influence on IAA content may be mediated by the up-regulation of *FZYs* and *PINs*. Further investigation into the underlying mechanism revealed that the expression levels of tomato *EXPs* were significantly affected by DADS. The expression levels of *EXPB2* and *beta-expansin precursor* were increased after 3 d, and those of *EXP1, EXPB3* and *EXLB1* were increased after 5 d of DADS treatment (0.41 mM). This result suggests that tomato root growth may be regulated by multiple expansin genes at different developmental stages. Therefore, we conclude that the effects of DADS on the root growth of tomato seedlings are likely caused by changes associated with cell division, phytohormones, and the expression levels of expansin genes.

## Introduction

Allelopathy is a widespread biological phenomenon by which one organism produces secondary metabolites that influence the growth, survival, development, and reproduction of other organisms. The use of allelopathic crops to control weeds, alleviate obstacles to continuous cropping or promote crop growth in agriculture is currently being tested (Cheema and Khaliq, 2000; Cheema et al., 2004; Yildirim and Guvenc, 2005; Wortman et al., 2013; Wezel et al., 2014; Cheng and Cheng, 2015). The allelopathic crop garlic, along with other plants of the genus *Allium*, are widely used in crop rotation and intercropping with many other crops, including vetiver (*Vetiveria zizanioides* L. Nash), pea (*Pisum sativum* L.), cotton (*Gossypium hirsutum* L.), geranium (*Pelargonium graveolens* L. Her.), cucumber (*Cucumis sativus* L.), tomato, eggplant (*Solanum melongena* L.), and pepper (*Capsicum annuum* L.), to improve the nutritional status of the soil, reduce plant diseases and insect pests, alleviate continuous cropping obstacles, and increase land use efficiency and net return (Babu et al., 1996; Marimuthu et al., 2013, 2014; Qasim et al., 2013; Singh et al., 2013; Xiao et al., 2013; Yaseen et al., 2014; Wang et al., 2015). The garlic-tomato intercropping system has been shown to improve microorganism populations and the enzyme activity of the medium, increase tomato fruit quality and produce a higher net income than a monoculture system (Liu T. J. et al., 2014). Zhou H. B. et al. (2013) reported that wheat-garlic intercropping or the application of a garlic oil blend or the garlic allelochemical diallyl disulfide (DADS) increased the population density of natural enemies (e.g., ladybeetles and mummified aphids) of the cereal aphid and the 1000-grain weight and yield of wheat.

Zhou et al. initially studied the allelopathy of garlic in 2007 and found that garlic root exudates had no observable effects on the germination rates, germination indices, or shoot heights of lettuce (*Lactuca sativa* L.), pepper, radish (*Raphanus sativus* L.), cucumber, Chinese cabbage (*Brassica pekinensis* L.), and tomato but significantly increased the root length, aboveground fresh mass, and root fresh mass of lettuce (Zhou et al., 2007). Garlic root exudates at low concentrations (0.1 and 0.2 g mL^−1^) have been demonstrated to promote lettuce seed germination and seedling growth, but high concentrations (0.4 and 0.6 g mL^−1^) have shown inhibitory effects (Zhou Y. L. et al., 2011). However, the allelopathic effect of DADS, the main allelochemical in garlic root exudates and garlic straw aqueous extracts (Jin et al., 2007; Liu et al., 2011; Zhou and Cheng, 2012), has not been studied.

DADS is a volatile organosulfur compound derived from garlic and a few other plants in the genus *Allium* (Kubota et al., 1999; Yin et al., 2003; Lanzotti et al., 2014). The steam-volatile components include diallyl, dimethyl, and allyl methyl sulfides, disulfides, and trisulfides as well as some other minor components, all of which are formed by the decomposition of allicin and are released by crushing garlic or other plants of the Alliaceae. These organosulfur compounds can inhibit carcinogen activation, boost detoxifying processes, cause cell cycle arrest (mostly in the G_2_/M phase), stimulate the mitochondrial apoptotic pathway and increase the acetylation of histones (Jo et al., 2008). Moreover, garlic-derived sulfur compounds can also influence gap-junctional intercellular communication and participate in the development of multidrug resistance (Iciek et al., 2009).

Numerous studies have revealed that garlic extract, volatile oil, allicin, and mercaptan sulfur compounds have strong antibacterial, antifungal, and antiviral activities (Ankri and Mirelman, 1999; Avato et al., 2000; Sealy et al., 2007; Casella et al., 2013; Mostafa et al., 2013). The antibacterial protective effect of garlic is due to its organosulfur compounds such as DADS, and this effect is enhanced by increased amounts of DADS (Avato et al., 2000; Tsao and Yin, 2001; Tsaoa and Yin, 2001). The antimicrobial properties of these compounds are thought to be based on their high membrane permeability and their participation in thiol-disulfide exchange reactions with the free thiol groups of proteins (Rabinkov et al., 1998; Miron et al., 2000).

Only a few studies about volatile garlic oil or DADS have focused on plants. Kubota et al. (1999) found that exposure to volatile diallyl di- and trisulfides was the most effective treatment for promoting the bud break of single-bud cuttings of “Kyoho” (*Vitis vinifera* × *labruscana* Bailey) irrespective of the concentration used and the duration of exposure. The active substances from garlic responsible for breaking bud dormancy in grapevines are sulfur-containing compounds containing an allyl group (CH_2_CHCH_2_), particularly DADS. Many researchers have demonstrated that garlic extracts or the sulfur-containing compounds of garlic can improve bud break in grapevines (Kubota et al., 2003; Potjanapimon et al., 2008; Vargas-Arispuro et al., 2008; Botelho et al., 2010). Garlic and onion extracts can hasten floral bud break and increase the bud break percentage, fruit set, the total number of fruits and the fruit yield of apple trees; onion extracts can also stimulate the accumulation of total indoles (precursors for auxin synthesis) in “Anna” apple fruits (Botelho and Muller, 2007; Perussi et al., 2010; El-Yazal and Rady, 2014; Rady and El-Yazal, 2014). In most of the research discussed above, there were multiple active substances present in the garlic root exudates and garlic oil, and they may function together as a mixture. However, the effects of the single substance DADS on plants have not been investigated.

The objective of this study was to investigate the physiological and biochemical effects of DADS on tomato seed germination and root growth. In our previous research, we found that this volatile allelochemical isolated from garlic seedlings and root exudates promoted growth of receiver plants at low concentrations but inhibited their growth at high concentrations (Zhou Y. L. et al., 2011). Moreover, different receptor plants showed different levels of sensitivity to these allelochemicals (Jin et al., 2007). Therefore, a wide range of DADS concentrations was used to investigate the allelopathic effects of DADS on tomato. In this study, seed germination and seedling growth, root elongation, cell division, phytohormone balance and the expression profile of the key auxin biosynthesis genes *FZY*s (*YUCs*), the auxin transport genes *SlPINs* and expansin genes (*EXPs*) in tomato root were investigated to determine the allelochemical effects of DADS on tomato. This research will deepen our understanding of the mechanism underlying the effect of DADS on seed germination and root growth in receiver plants and will lay the theoretical foundation for the application of DADS in agricultural production.

## Materials and methods

### Materials

Tomato (*Solanum lycopersicum* L. var. Dongfen No. 3) seeds were bought from the Yufeng Seed Company (Yangling, China). DADS was purchased from Fluka Chemika Co. (Bucha, Switzerland). DADS and Tween-80 were dissolved at a ratio of 1:2 (v:w) and stored at 4°C after dilution to 20.67 mM as the stock solution (Zhou Y. et al., 2013). Distilled water was used for a water control group, and the maximum amount of Tween-80 (0.6%) was used for the solvent control group.

### Germination of tomato seeds and seedling growth in Petri dishes

Fifty tomato seeds were sorted by size and evenly placed on two layers of filter paper in a closed, sterile Petri dish (11 cm in diameter and 7 cm high with a vent in the lid). Initially, 6 mL of DADS solution at one of eleven concentrations (0.01, 0.04, 0.10, 0.21, 0.41, 0.62, 1.45, 2.89, 6.20, 12.40, or 20.67 mM) was added to each Petri dish, with equal volumes of distilled water and Tween-80 (0.6%) used separately as controls. Subsequently, 2 mL of DADS solution was added at the same time every other day to replenish the solution that had been consumed. The Petri dishes were placed in a growth chamber (25 ± 1°C) in continuous darkness and exposed to light (white light with an illuminance of 60 μmol m^−2^ s^−1^) when the germination rate reached 80%. Treatments were arranged in a completely randomized design with four replications. Germination was determined by counting the number of germinated seeds at 24-h intervals over a 7-day period. The lengths of the roots and hypocotyls and the fresh weights of the roots and the aerial parts were measured on the 14th day.

### Relative root elongation (RRE) of tomato seedlings after DADS treatment

Tomato seed germination was accelerated after hot water treatment. Rectangular Petri dishes (19 cm long × 13 cm wide × 12 cm tall) with a layer of filter paper in the bottom were used when the length of the radicle reached 5 mm. The germinated seeds were placed between the bottom of the dish and the filter paper. The Petri dishes were tilted at an angle of 15 degrees. DADS solution, 8 mL of one of eleven concentrations (0.01, 0.04, 0.10, 0.21, 0.41, 0.62, 1.45, 2.89, 6.20, 12.40, and 20.67 mM), was added to the bottom of the dish to soak the filter paper completely. Then, 3 mL of DADS solution was added every other day to replace the solution that had been consumed. Root length was measured at 24-h intervals over a 5-day period. Relative root elongation (% greater than the water control) was determined using the following equation: (ΔT/ΔCK-1) × 100 (Yang et al., 2010). ΔT represents the increase in root length after every 24 h of DADS treatment or in the solvent control (Tween-80). ΔCK represents the increase in root length every 24 h in the water control. Experimental treatments were replicated three times, and 24 tomato roots were measured in each experiment.

### Mitotic index of root tips

The mitotic index of root tips was measured using the traditional squash technique with carbol fuchsin staining. After 24 or 48 h of DADS (0, 0.04, 0.21, 0.41, 1.45, 6.20, and 12.40 mM) treatment, distal fragments of root tips (8 mm long) were cut off and fixed in Carnoy's fixative (glacial acetic acid:ethanol, 3:1) for 30 min. Then, the fragments were washed three times and placed in 70% ethanol at 4°C. The preserved root fragments were hydrolyzed in 0.1 M HCl at 60°C for 20 min, washed three times, and then stained with carbol fuchsin (0.3% basic fuchsin, 5% phenol in distilled water, w/v) for 30 min and transferred onto a slide. Meristems were cut off and squashed under a coverslip to separate the cells. Cells were observed using a light microscope (Olympus PROVIS AX70) under 400 × magnification, and 10,000 cells were analyzed for each treatment to determine the numbers of cells in each phase of mitosis. The results are expressed as percentages. Ten root tips were counted for each treatment.

### The sizes of the meristem and meristematic cells in tomato roots

The sizes of the meristem and meristematic cells were measured by preparation of conventional paraffin sections. The sampling method was the same as that used for the measurement of the mitotic index. Root samples were fixed in formalin-acetic acid-alcohol (FAA) (formaldehyde:acetate:70% alcohol, 1:1:18) and then placed at 4°C. Fixed samples were dehydrated in a graded series of ethanol (70, 80, 90, 95, 100% for 20 min each) followed by an ethanol/xylene series (ethanol:xylene 2:1, ethanol:xylene 1:2, 100% xylene). Xylene was gradually replaced with paraffin at 60°C overnight in an open jar to allow traces of xylene to evaporate (two changes of paraffin for 2 h each), and samples were subsequently embedded in paraffin. The samples were sectioned longitudinally using a rotary microtome (Leica RM2016), and the 7-μm-thick paraffin ribbon was placed and stretched out in a water bath at 42°C. Sections were mounted onto slides, air dried for 30 min and then baked in a 42°C oven overnight. For staining, the slides were deparaffinized in xylene for 10 min (twice), then rehydrated in a graded series of ethanol (100 and 95%), and stained with 1% safranin followed by 0.1% Fast Green FCF. The stained sections were dehydrated in the graded ethanol series and xylene and then mounted with Canada balsam neutral mounting medium. Photographs of root segments were obtained using an Olympus PROVIS AX70 microscope with the programs View Finder and Studio Lite (Olympus). All the data were counted using ImageJ software. The sizes of the meristematic cells were measured as the widths and lengths of isodiametric cells of the endodermis in the root meristem. The meristematic length was determined as the distance between the apical meristem and the first square cells in the meristem (Yang et al., 2010). The meristematic width was measured as the widest part of the meristem. Experiments were repeated twice, and 10 root tips were evaluated for each treatment.

### Determination of levels of plant hormones

The extraction, purification and determination of endogenous levels of IAA (indole-3-acetic acid), gibberellic acid (GA), and ZR (zeatin riboside) were performed using an indirect enzyme-linked immunosorbent assay (ELISA) technique following previously described methods (Chen et al., 1997; Yang et al., 2001) with some modifications. The roots of 14-day-old seedlings cultured in water or DADS solutions were ground in a mortar on ice with 10 mL of 80% (v/v) methanol extraction medium containing 1 mM butylated hydroxytoluene as an antioxidant. The extract was incubated at 4°C for 4 h and centrifuged at 10,000 × g for 10 min at 4°C. The supernatants were passed through Chromosep C18 columns (C18 Sep-Pak cartridge; Waters Corp., Milford, MA, USA) that had been prewashed with 5 mL methanol (100%), 5 mL diethyl ether (100%), 5 mL methanol (100%), and 1 mL of 80% methanol. The hormone fractions were dried under N_2_ and then each dissolved in 1 mL of phosphate-buffered saline containing 0.1% (v/v) Tween-20 and 0.1% (w/v) gelatin (pH 7.5). The IAA, GA and ZR contents were determined using previously described ELISA methods (Yang et al., 2001). Microtitration plates (Nunc, Roskilde, Denmark) were coated with synthetic IAA-, GA,- or ZR-ovalbumin conjugates in NaHCO_3_ buffer (50 mmol L^−1^, pH 9.6) and incubated at 37°C overnight. Ovalbumin solution (10 mg mL^−1^) was added to each well to block nonspecific binding. After incubation for 30 min at 37°C, IAA, GA, and ZR standards, sample extracts and antibodies were added, and the plate was incubated for another 45 min at 37°C. The antibodies against IAA, GA, and ZR were obtained as described previously (Weiler et al., 1981). Then, horseradish peroxidase-labeled goat anti-rabbit immunoglobulin was added to each well, and the plate was incubated at 37°C for 1 h. Finally, the buffered enzyme substrate (orthophenylenediamine) was added, and the enzyme reaction was carried out in the dark at 37°C for 15 min and then terminated using 3 M H_2_SO_4_. The absorbance at 490 nm was recorded. Analysis of the ELISA data was performed as described by Weiler in 1981 (Weiler et al., 1981). In the current study, the percentage of recovery of each hormone was calculated by adding known quantities of standards to a split extract. All the recovery percentages were >90%, and all the sample extract dilution curves paralleled the standard curves, indicating that there were no nonspecific inhibitors in the extracts. Each hormone was assayed in triplicate.

### Tomato seedling root hair length and density

Tomato seeds were treated with DADS or distilled water for 48 h after sprouting. Root segments (5 mm) were excised from each seedling at 0.5, 1.5, and 2.5 cm behind the root tip and then fixed in 70% ethanol. The fixed root segments were placed on slides, and each segment was immersed in a drop of water. Root hairs were observed under a light microscope (Olympus PROVIS AX70) using the programs View Finder and Studio Lite (Olympus).

### Expression of auxin biosynthesis genes (*FZY*s), auxin transport genes (*SlPINs*), and expansin genes (*EXPs*)

The expression profiles of *FZY* and *SlPIN* genes in tomato root were evaluated in tomato seedlings treated with different concentrations of DADS for 24 h. The expression profiles of *EXP* genes were evaluated in tomato seedlings treated with 0.41 mM DADS for 1, 3, and 5 d. The total RNA of tomato roots was isolated using the Column Plant RNAout (Tiandz Inc., Beijing) according to the manufacturer's instructions and then treated with RNase-free DNase I (Promega, Madison, WI, USA) to remove the contaminating genomic DNA. Reverse transcription (RT) was carried out using the RevertAid RT Reverse Transcription kit (Thermo Fisher Scientific) according to the manufacturer's instructions. The cDNA was used for real-time fluorescent quantitative PCR (qPCR) with specific primers. The primers for the *EXPs, FZYs*, and *SlPINs* (Table S1) were designed using Primer3 and BLAST, and the specificity of each primer for its corresponding gene was verified. To normalize the total amount of cDNA present in each reaction, the tomato *Actin-2/7* gene was used as an internal control for calibration of relative expression (Table S1; de Jong et al., 2009). The qPCR was performed with the Maxima SYBR Green qPCR Master Mix (Thermo Fisher Scientific) following the manufacturer's recommendations and using a Real-time qPCR Detection System (iQ5, Bio-Rad, USA). Data were analyzed using the 2^−Δ*ΔCT*^ method (Livak and Schmittgen, 2001) and are presented as the relative levels of gene expression, which were calculated relative to the water control. For each gene analysis, three biological replicates were performed.

### Statistical analysis

All data were analyzed using SAS (SAS Institute, Cary, NC, USA). Mean values were computed for each experiment and are reported with their standard errors (SE) to indicate the variation associated with each particular mean value. Fisher's Least Significant Difference (LSD) test or Student's *t*-test was used to detect differences between treatments with different concentrations of DADS and the water control.

## Results

### Influence of DADS on tomato seed germination and seedling growth

**Figure 1:**
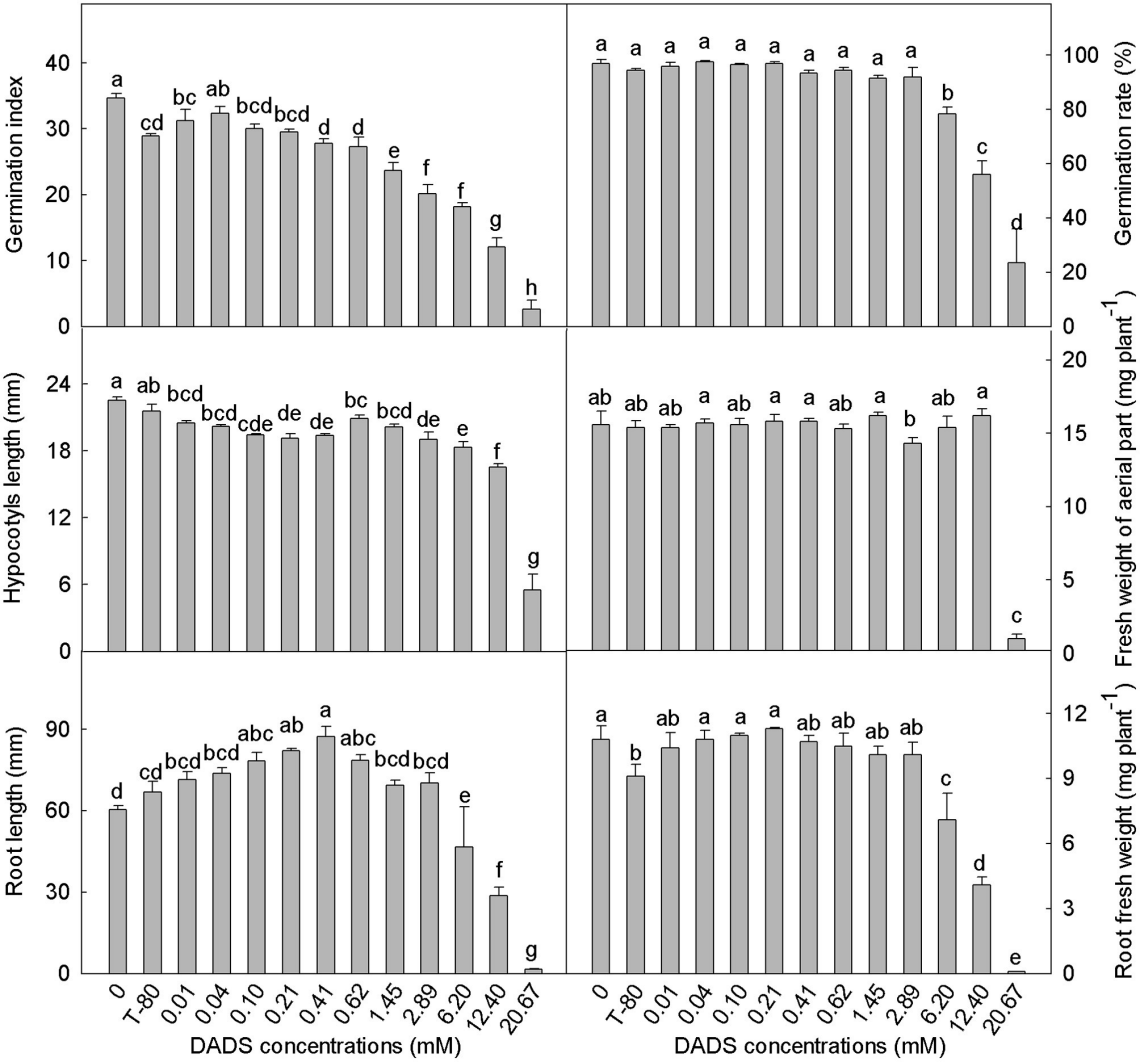
**Effects of different concentrations of DADS on tomato seed germination and seedling growth**. Germination index and germination rate were measured 7 d after DADS treatment, *N* = 4; hypocotyl length, fresh weight of aerial part, root length, and root fresh weight were measured 14 d after DADS treatment, *N* = 40 (LSD test). Bars with different letters are significantly different at the 0.05 level.

**Figure 2:**
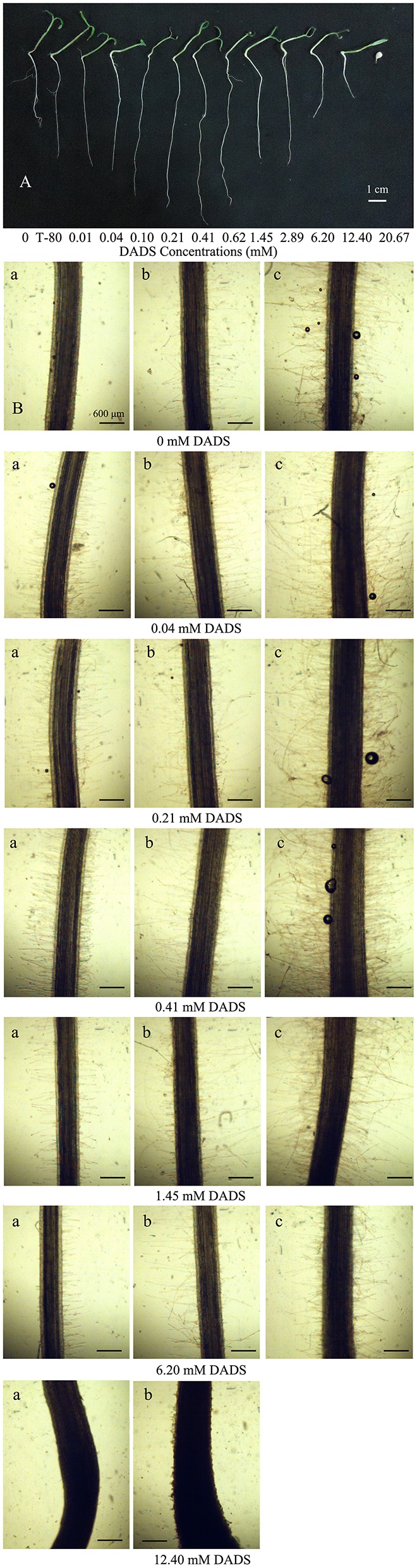
**Effect of increasing concentrations of DADS on tomato root development. (A)** Tomato root length influenced by DADS. Tomato seedlings grew for 14 d in water (control), T-80 (Tween-80, 0.6%) or DADS aqueous solutions (*Bar* = 1 cm). **(B)** Length and density of tomato root hairs affected by DADS. Tomato seedlings treated with DADS aqueous solutions for 48 h. (a) Root segments (5 mm) excised from seedling at 0.5 cm behind the root tip; (b) Root segments (5 mm) excised from seedling at 1.5 cm behind the root tip; (c) Root segments (5 mm) excised from seedling at 2.5 cm behind the root tip (*Bar* = 600 μm).

The tomato seed germination index was generally reduced by DADS treatment (Figure 1). Low concentrations of DADS did not affect the germination rate, but high concentrations (6.20–20.67 mM) of DADS significantly reduced the germination rate. Hypocotyl length was decreased by DADS at all concentrations compared with the water and solvent controls. However, the fresh weights of tomato hypocotyls treated with DADS were not affected, except at concentrations of 2.89 and 20.67 mM. As shown in Figures 1, 2A, the results reflect a trend of increasing tomato root length at low concentrations but a decreasing trend at high concentrations of DADS. Root length reached a maximum of 87.4 mm at the concentration of 0.41 mM DADS. In addition, low levels of DADS did not affect the root fresh weight, but high concentrations significantly reduced root fresh weight. These results suggest that DADS did not affect tomato seed germination at low concentrations (0.01–0.62 mM) but significantly inhibited it at high concentrations (6.20–20.67 mM). In contrast, seedling growth was promoted by DADS at low concentrations (0.01–0.62 mM) but inhibited at high concentrations (6.20–20.67 mM).

### Influence of DADS on the RRE of tomato

The effect of DADS on the RRE of tomato is summarized in Table 1. The solvent control promoted tomato root growth in the initial period of the experiment (1–2 d) but inhibited it from 3 to 5 d. The RRE of tomato was greater than that of the water control at low concentrations (0.01–1.45 mM) of DADS during the entire length of the treatment. Moreover, the RRE of tomato was significantly higher when treated with 0.04 or 0.41 mM DADS than for the solvent control during the whole process (1–5 d). However, the RRE of tomato seedlings treated with 2.89 mM DADS increased during the first 2 days, reached 26.85% on the second day, and gradually decreased during the last 3 days. In contrast, the treatments with high levels of DADS (12.40 and 20.67 mM) strongly decreased root growth compared with the water control group during the treatment process. These results suggest that DADS increases the RRE of tomato at low concentrations (0.01–0.62 mM) but decreases it at high concentrations (6.20–20.67 mM).

**Table 1:** **Relative root elongation (RRE) of tomato seeding from 1 to 5 d after different levels of DADS treatment**.

**DADS concentration (mM)**	**Tomato RRE (% greater than the water control)**				
	**Days for DADS treatment**				
	**1**	**2**	**3**	**4**	**5**
T-80	−14.26 ± 1.08d	−2.87 ± 0.51c	13.37 ± 3.41ef	12.77 ± 3.64bc	17.65 ± 5.53b
0.01	**22.55** ± **5.49a**	**7.05** ± **1.19bc**	**22.72** ± **4.62cde**	**12.53** ± **0.17bc**	**19.46** ± **4.02b**
0.04	**19.54** ± **2.06a**	**24.44** ± **2.64a**	**36.91** ± **5.57a**	**38.16** ± **7.52a**	**38.90** ± **7.44a**
0.10	**21.65** ± **3.49a**	**17.96** ± **7.80ab**	**33.84** ± **2.54ab**	**36.96** ± **5.54a**	**11.87** ± **4.32bc**
0.21	**19.16** ± **4.34a**	**20.80** ± **6.13a**	**24.37** ± **1.62bcd**	**7.91** ± **0.68c**	**12.54** ± **5.90bc**
0.41	**9.31** ± **0.74b**	**17.87** ± **8.28ab**	**32.36** ± **5.57abc**	**36.73** ± **4.50a**	**35.91** ± **3.88a**
0.62	**5.77** ± **0.68b**	**18.74** ± **8.04ab**	**9.30** ± **0.51f**	**16.55** ± **6.25bc**	**25.36** ± **8.22ab**
1.45	**2.50** ± **1.27bc**	**15.39** ± **7.81ab**	**11.61** ± **4.15f**	**19.07** ± **0.80b**	**14.61** ± **6.25bc**
2.89	−2.14 ± 2.78c	26.85 ± 3.37a	8.65 ± 2.61f	7.81 ± 5.71c	2.35 ± 0.67cd
6.20	−23.70 ± 2.51e	19.46 ± 5.51ab	15.61 ± 6.40def	−11.00 ± 5.17d	−3.15 ± 0.12d
12.40	−**82.44** ± **1.78f**	−**78.01** ± **3.21d**	−**66.15** ± **4.40g**	−**83.84** ± **1.72e**	−**77.28** ± **2.49e**
20.67	−**94.53** ± **0.59g**	−**98.59** ± **0.69e**	−**97.95** ± **2.05h**	−**99.39** ± **0.61f**	−**100.00** ± **0.00 f**

*Tomato RRE determined as root length increase% everyday. The positive value (>0) means RRE is higher than that in the water control group; negative value (< 0) means RRE is lower than that in the water control group. Values for particular treatment (in columns, mean ± SE) followed by different small letters are significantly different at the 0.05 level, N = 24 (LSD test). The bold values represent the important differences between treatments and the water control*.

### DADS induces alterations in the mitotic activity of tomato root tip cells

The mitotic index of the root tips changed gradually after treatment with DADS, and this change was concentration dependent (Table 2). After 24 h of culture with 0.21 mM DADS, the percentage of dividing cells (5.01%) was significantly higher than in the water control (4.38%). During prolonged (48 h) DADS treatment, the mitotic indices were increased at low concentrations of DADS (0.04–0.41 mM), although there was no significant difference compared with the water control. Additionally, the strongest inhibitory effect was observed in roots cultured in 12.40 mM DADS; the mitotic index was unchanged over the 2 days, remaining at ~2.35%.

**Table 2:** **Mitotic index and mitotic phases of tomato root cells exposed to different concentrations of DADS after 24 and 48 h**.

	**DADS concentration (mM)**	**Mitotic index (%)**	**Dividing cells in different phases of mitosis**			
			**Prophase (%)**	**Metaphase (%)**	**Anaphase (%)**	**Telophase (%)**
24 h	0	4.38 ± 0.24b	3.04 ± 0.21a	0.90 ± 0.06bc	0.19 ± 0.04ab	0.25 ± 0.06ab
	0.04	4.09 ± 0.21b	2.61 ± 0.14b	1.04 ± 0.09b	0.21 ± 0.02a	0.23 ± 0.04ab
	0.21	**5.01** ± **0.21a**	3.26 ± 0.13a	**1.33** ± **0.10a**	0.18 ± 0.04ab	0.23 ± 0.07ab
	0.41	4.62 ± 0.22ab	3.15 ± 0.19a	1.05 ± 0.08b	0.12 ± 0.03bc	0.30 ± 0.04a
	1.45	3.24 ± 0.11c	2.36 ± 0.10bc	0.72 ± 0.06c	0.08 ± 0.02c	0.09 ± 0.02c
	6.20	3.09 ± 0.18c	2.08 ± 0.11cd	0.71 ± 0.07c	0.14 ± 0.02abc	0.16 ± 0.03bc
	12.40	**2.34** ± **0.14d**	**1.80** ± **0.09d**	**0.39** ± **0.08d**	**0.09** ± **0.02c**	**0.07** ± **0.03c**
48 h	0	3.81 ± 0.24ab	2.74 ± 0.29ab	0.78 ± 0.06b	0.11 ± 0.02b	0.19 ± 0.05ab
	0.04	4.03 ± 0.25a	2.71 ± 0.17ab	**1.04** ± **0.09a**	0.12 ± 0.03b	0.16 ± 0.04abc
	0.21	4.16 ± 0.22a	**2.85** ± **0.14a**	0.88 ± 0.07ab	**0.21** ± **0.04a**	0.23 ± 0.02ab
	0.41	4.12 ± 0.21a	2.66 ± 0.15ab	**1.07** ± **0.07a**	0.13 ± 0.02b	0.26 ± 0.05a
	1.45	3.33 ± 0.22bc	2.31 ± 0.12bc	0.77 ± 0.09b	0.12 ± 0.03b	0.13 ± 0.03bc
	6.20	3.11 ± 0.16c	2.15 ± 0.09cd	0.70 ± 0.05bc	0.13 ± 0.02b	0.14 ± 0.03bc
	12.40	**2.35** ± **0.22d**	1.68 ± 0.18d	0.50 ± 0.06c	0.11 ± 0.02b	0.06 ± 0.02c

*Mitotic index and frequencies of each mitotic phase were calculated for 10,000 cells. Values for particular treatment (in columns, mean ± SE) followed by different small letters are significantly different at the 0.05 level, N = 10 (LSD test). The bold values represent the important differences between treatments and the water control*.

How DADS modified the proportions of mitotic phases in tomato root tip cells varied over 48 h (Table 2). When exposed to DADS for 24 h, dividing cells in metaphase were increased significantly by 0.21 mM DADS, whereas the cells in telophase were increased by 0.41 mM DADS. At 48 h of treatment with 0.21 mM DADS, the proportion of cells in anaphase was significantly increased. DADS concentrations of 0.04 and 0.41 mM also strongly increased the proportion of cells in metaphase after 48 h. Dividing cells in metaphase were increased after both 24 and 48 h in the 0.41 mM DADS condition, but there was no significant difference at 24 h compared with the water control. Just like the mitotic index, DADS treatment at 12.40 mM for 24 h significantly decreased the proportions of cells in all phases of mitosis. Overall, low levels of DADS (0.04–0.41 mM) increased tomato root mitotic activity, whereas DADS concentrations between 1.45 and 12.40 mM decreased it.

### DADS induces alterations in the sizes of the root meristem and meristematic cells

**Figure 3:**
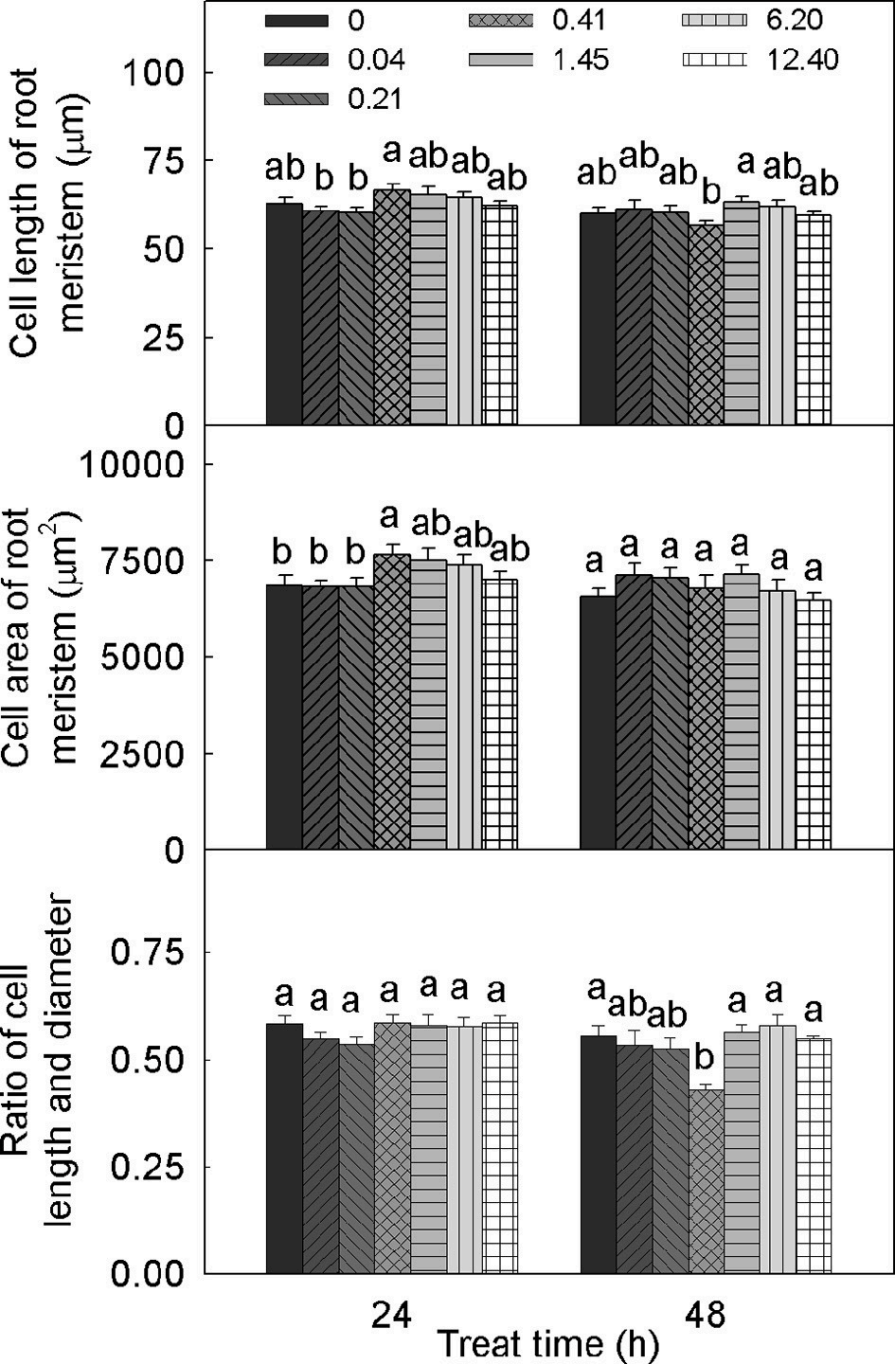
**Meristem cell size of tomato root after treatment with DADS at various concentrations (24 and 48 h)**. Bars with different letters are significantly different at the 0.05 level (LSD test, *N* = 10).

The different DADS concentrations and treatment times had different effects on cell length in tomato root meristem, but DADS treatment produced no significant differences compared with the water control (Figure 3). When treated for 24 h with 0.41 mM DADS, cell lengths in tomato root meristem reached a maximum of 66.6 μm but achieved a minimum of 56.7 μm after 48 h. There were no obvious differences in the cell widths of the root meristems among the treated seedlings (data not shown). Treatment with 0.41 mM DADS for 24 h significantly increased the cell area of the root meristem. No significant differences in the ratios of cell length to diameter were observed except for a decreased ratio in tomato roots after 48 h of treatment with 0.41 mM DADS.

**Figure 4:**
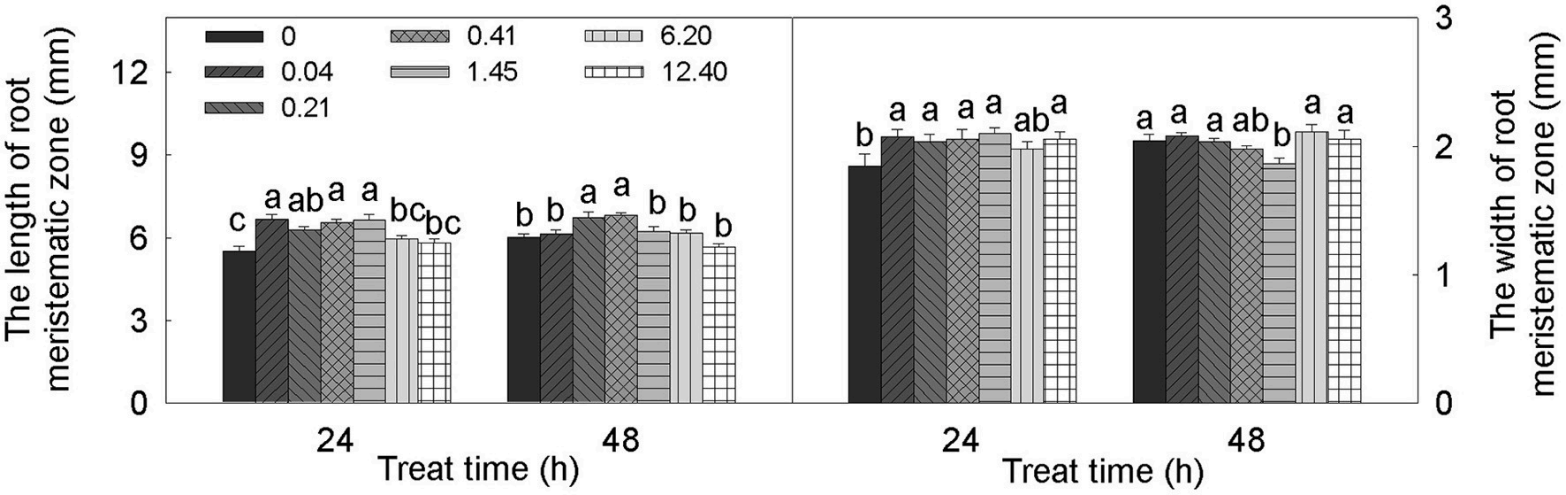
**Meristem length and width of tomato root after treatment with DADS at various concentrations (24 and 48 h)**. Bars with different letters are significantly different at the 0.05 level (LSD test, *N* = 10).

The alteration of root meristem size by DADS treatment was concentration dependent (Figure 4). Over 24 h, treatment with DADS concentrations of 0.04–1.45 mM significantly increased the length of the meristematic zone. In addition, the growth-promoting effect of DADS at concentrations of 0.21 and 0.41 mM lasted 48 h. The width of the meristematic zone treated with DADS for 24 h was significantly increased except by treatment with DADS at 6.20 mM. However, this growth-promoting effect weakened over time, until the cell width increase was similar to the increase observed in the water control after 48 h. These results suggest that relatively low concentrations (0.04–1.45 mM) of DADS can enlarge meristem size after a short treatment time.

### Effects of DADS on root hair density and length

The influence of DADS on tomato root hairs is shown in Figure 2B. When the concentration of DADS was increased from 0.04 to 0.41 mM, the length and density of root hairs at 0.5 and 2.5 cm behind the root tip were increased. In contrast, root hair length and density were decreased by treatment with 1.45–12.40 mM DADS. A very short root-hair length was found in tomato roots that reached only 2.0 cm in length after treatment with 12.40 mM DADS for 2 d (Figure 2B). This suggests that tomato root hair density and length were increased by DADS at low concentrations (0.04–0.41 mM) but decreased at high concentrations (1.45–12.40 mM).

### DADS alters phytohormone homeostasis in tomato roots

**Figure 5:**
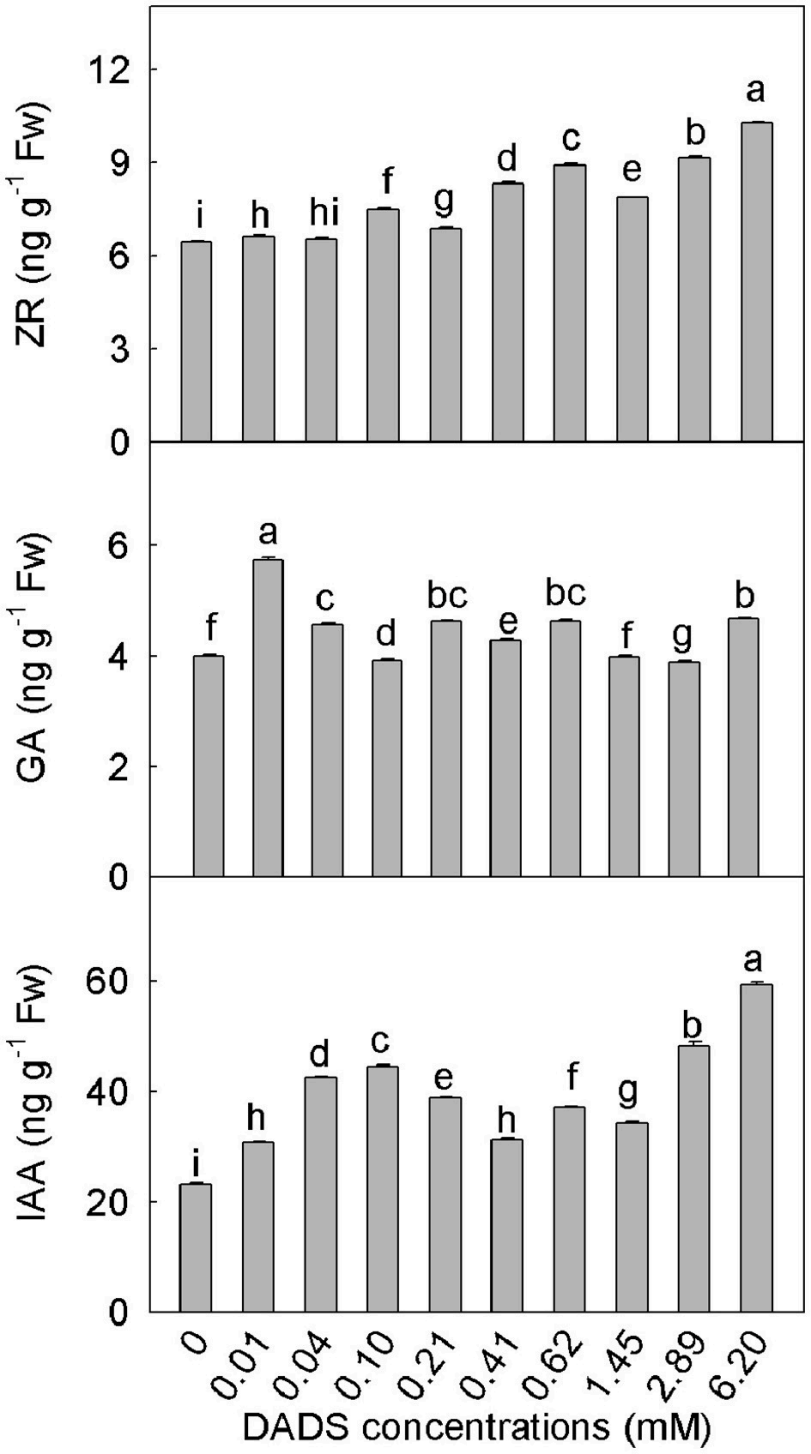
**Contents of ZR, GA, and IAA in tomato roots after application of DADS for 14 d**. Bars with different letters are significantly different according to the LSD test at the 0.05 level, *N* = 3.

DADS had different effects on different phytohormones (Figure 5). After DADS treatment, the content of ZR in tomato roots was significantly greater than that in the water control (with the exception of treatment with 0.04 mM DADS), and the ZR content increased in a dose-dependent manner. At the maximum concentration of DADS (6.20 mM), the accumulation of ZR reached a maximum of 10.27 ng g^−1^ fresh weight, which was approximately 0.6 times greater than the level of ZR in the water control. The levels of GA varied with the different DADS concentrations, but the extent of variation was less than for ZR. Except for treatments with 1.45 and 2.89 mM DADS, the GA contents of tomato roots were obviously increased. However, the GA content was greatest at the lowest concentration of DADS (0.01 mM), at which the GA level was approximately 1.4 times the level in the water control. The lowest level of GA content was observed at 2.89 mM DADS. As shown in Figure 5, various concentrations of DADS increased the IAA content. Similar to ZR, IAA content was highest after treatment with 6.20 mM DADS, at 59.43 ng g^−1^ fresh weight, which was 1.6 times greater than that in the water control.

### DADS influences the expression of auxin biosynthesis genes (*FZY*s) and auxin transport genes (*SlPINs*) in tomato roots

**Figure 6:**
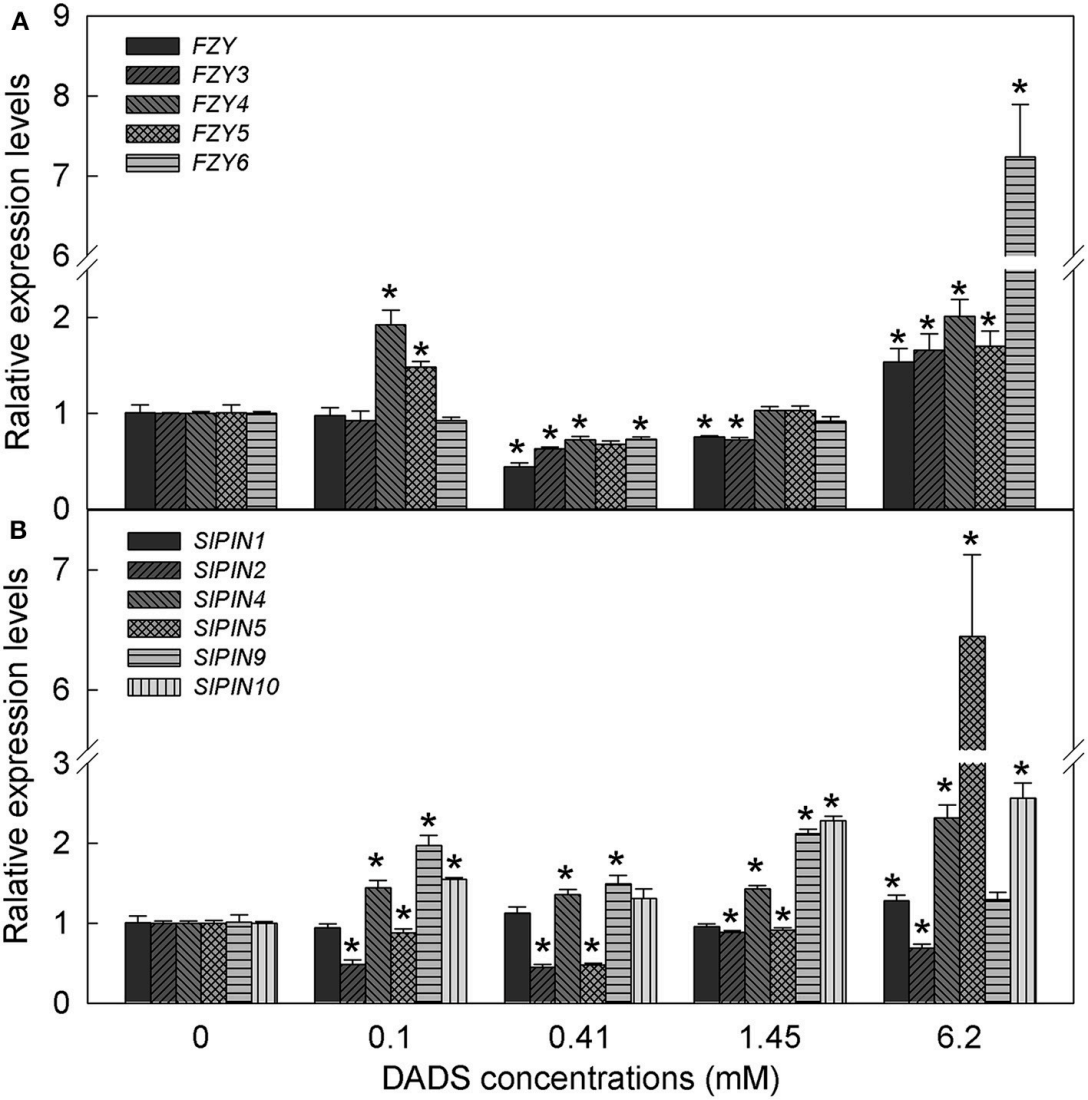
**Relative expression levels of ***FZY*** and ***SlPIN*** genes in tomato root after treatment with DADS at various concentrations for 24 h. (A)** Expression levels of *FZY* genes. **(B)** Expression levels of *SlPIN* genes. ^*^Significant differences according to the *T* test (*P* = 0.05), *N* = 3.

Based on the IAA content in tomato root, we hypothesized that IAA might play a major role in DADS-regulated root growth of tomato seedlings. Therefore, the expression profiles of auxin biosynthesis genes (*FZYs*) and auxin transport genes (*SlPINs*) in tomato root were analyzed under different concentrations of DADS. After treatment with 0.1 mM DADS, the expression levels of *FZY4, FZY5, SlPIN4, SlPIN9*, and *SlPIN10* increased significantly (Figure 6), as did the IAA content (Figure 5). With increasing DADS concentrations (0.41–1.45 mM), the expression levels of *FZYs* were significantly lower or showed no notable difference compared with the control, while the transcript levels of *SlPIN4, SlPIN9*, and *SlPIN10* were higher than those in the control (Figure 6). IAA content showed a decreasing trend at these DADS concentrations (Figure 5). When the DADS concentration reached 6.2 mM, except for *SlPIN2*, the expression levels of *FZYs* and *SlPINs* were higher than in the control (Figure 6), and the IAA content was also the highest at this DADS concentration (Figure 5).

### DADS modifies the expression of expansin genes (*EXPs*)

**Figure 7:**
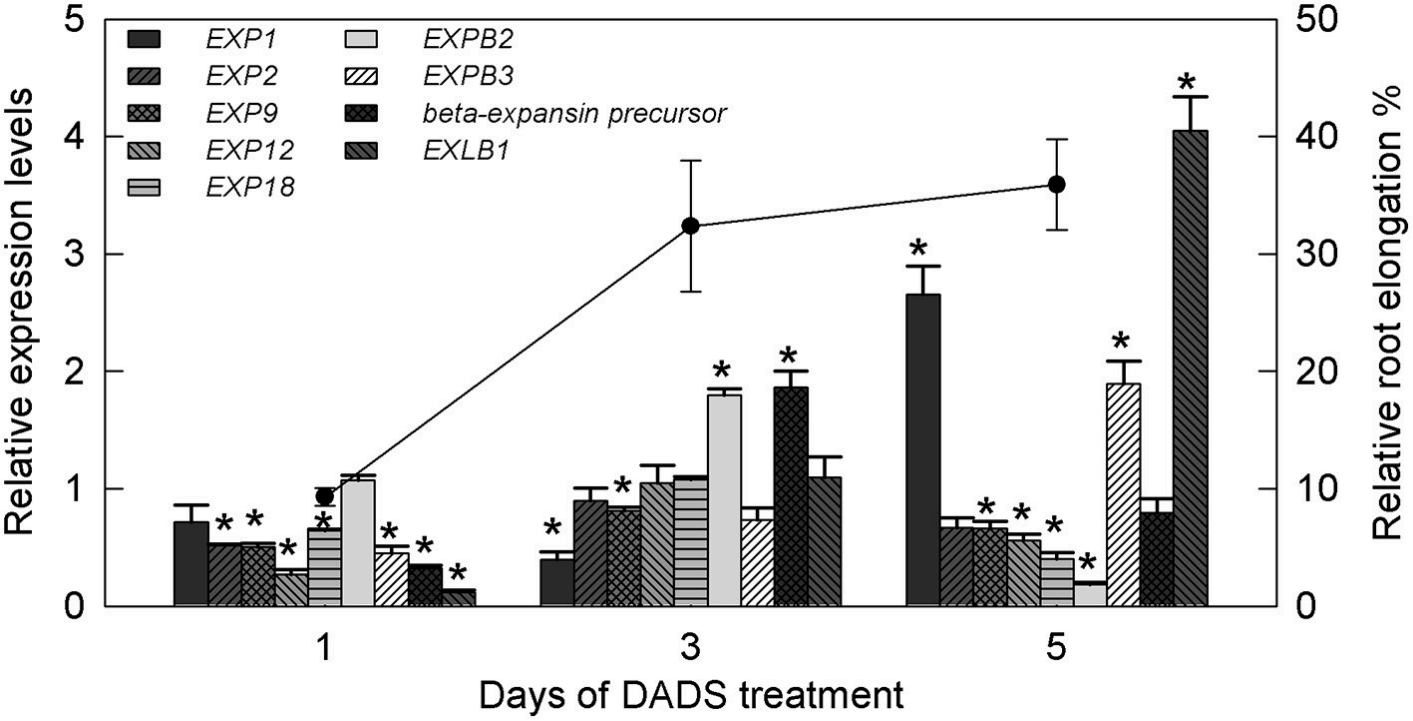
**Relative transcript levels of expansin genes in tomato root and relative root elongation after treatment with 0.41 mM DADS**. Histogram shows the relative transcript level of expansin genes. A value of 1 means the expression level is equal to that in the control. ^*^Significant differences according to the *T*-test (*P* = 0.05), *N* = 3. Line graph represents relative root elongation (% greater than the water control).

Based on the above results, the most significant effect of DADS in promoting tomato root growth was observed at the concentration of 0.41 mM. Therefore, this concentration was used for further research on the expression of tomato expansin genes (*EXPs*). The relative expression levels of *EXPs* in the water controls and DADS (0.41 mM)-treated tomato seedling roots at 1, 3, and 5 d are shown in Figure 7. The transcript abundance of *EXP1* was unchanged on day 1, significantly lower than in the water control on day 3, and significantly higher on day 5. The relative expression of *EXP2* was markedly lower than in the water control on day 1 but showed no differences on days 3 and 5. The mRNA level of *EXP9* was significantly lower than in the water control during treatment. Among all analyzed genes, two (*EXP12* and *EXP18*) were significantly inhibited at days 1 and 5 but showed no effect on day 3. In contrast with the expression of *EXP1*, that of *EXPB2* was upregulated on day 3 but was strongly downregulated on day 5 compared with the water control. Upon treatment with DADS, the expression level of *beta-expansin precursor* showed a decrease, an increase and an eventual return to its normal level. The expression profiles of *EXPB3* and *EXPB1* were of special interest, showing increases from 0.447 to 0.118 (1 d) to 1.90 and 4.05 (5 d), respectively. Meanwhile, the RRE of tomato was greatly increased during the duration of the 0.41 mM DADS treatment and was 9.31% (1 d), 32.36% (3 d), and 35.91% (5 d) higher than in the water control (Figure 7).

## Discussion

Most allelochemicals, which are non-nutritive substances produced mainly as plant secondary metabolites or as decomposition products of microbes, are highly phytotoxic to receivers. They can cause changes in the microstructure and ultrastructure of cells, inhibit cell division and elongation, alter phytohormone levels, and influence respiration, photosynthesis and gene expression (Lin et al., 2001; Singh et al., 2003; Sánchez-Moreiras et al., 2008). Commonly cited effects of allelopathy include reductions in seed germination, root and hypocotyl growth, cell elongation, and cell division of seedlings (Cruz Ortega et al., 1988; Turker et al., 2008; Iqbal and Fry, 2012). When the seeds of susceptible plant species are exposed to allelochemicals, germination may be inhibited, and the seedlings may show abnormal growth, development and metabolism. The most visible observable effects are retarded germination, short or no roots, lack of root hairs, abnormally long or short shoots, swollen seeds, and low reproductive ability (Rice, 1984; Soltys et al., 2011, 2012, 2014). However, in many cases, it has been reported that the responses to allelochemicals demonstrate stimulation at low concentrations and inhibition at high concentrations (Fischer, 1986; Liu de and An, 2005; Belz, 2008; Hong et al., 2008). Plant growth may be stimulated below the allelopathic threshold, but severe growth reductions may be observed above the threshold concentration, which is dependent on the sensitivity of the receiving species (Chon and Nelson, 2010).

According to all the results of the present study, the concentrations of DADS can be divided into three ranges: low concentrations (0.01–0.62 mM), medium concentrations (1.45–2.89 mM), and high concentrations (6.20–20.67 mM). In this study, compared with the solvent control, the root length and fresh weights of the aerial parts and roots were increased by DADS at low concentrations but inhibited at high concentrations (Figures 1, 2A). Dose-dependent effects of DADS on the RRE of tomato and root hair density and length were also observed in response to the tested concentrations compared with the water control group (Table 1 and Figure 2B). Similar effects on plant growth after treatment with particular allelochemicals have previously been detected by many researchers (Prithiviraj et al., 2007; Wang et al., 2009; Rashid and Reshi, 2012; Yang et al., 2013). Moreover, DADS treatment at low concentrations did not affect the germination rate of tomato seeds, possibly because the low concentrations tested are below the minimum inhibitory concentration (MIC) of DADS on tomato, whereas the inhibition effect observed at high concentrations of DADS may have been caused by the function of DADS and its osmotic effects. The different effects of DADS on tomato roots and shoots may due to the varying sensitivities of different plant tissues to DADS.

In our experiments, tomato root growth was significantly affected by DADS. Cell division and elongation in seedling roots are two essential processes for root growth and development. Cell division, which is initiated in meristems, provides new cells that are subsequently amplified and modified by cell enlargement within the elongation zone (Cosgrove, 1997). Inhibition of mitosis leads to reduced root length, even without affecting cell enlargement (Soltys et al., 2012). The effects of DADS on tomato root growth may be partly caused by the promotion/suppression of cell division. In addition, different alterations in the frequency of each phase of mitosis imply that the phases of mitosis have different sensitivities to DADS. There are many examples of allelochemicals inducing growth promotion or restriction, reflected in the root mitotic index. In a previous study, Ding et al. (2010) found that the allelochemical rabdosin B significantly increased the lettuce root mitotic index at lower levels (20–80 μM) but decreased it at higher concentrations (120–200 μM). Comparable results were obtained in our study. For most allelochemicals, plants have adapted to small amounts of the phytotoxins and can alleviate the toxic effects by themselves when the dose is within the MIC. However, inhibitory effects were observed when the concentrations of the allelochemicals tested were beyond the MIC. A promotion effect may be observed if the applied doses of the allelochemical are at certain low levels.

Root tip sizes are closely associated with the numbers and sizes of mitotic cells. In our study, the cell length of meristematic cells was not significantly affected by DADS, whereas the length of the meristematic zone was increased by DADS at low concentrations (Figures 3, 4). This result suggests that DADS stimulated mitosis and thus increased the cell numbers in the meristem and enlarged the length of the meristematic zone in the root. In addition, the width of the meristematic area was increased during all the 1-day DADS treatments, which implies that DADS can promote radial cell division in the root tip during a short treatment. Similarly, Yang et al. (2013) found that the promotion of root growth by lower levels of Gla A and B (20–40 μM) resulted from an increased mature cell length and from increased mitotic activity. We have demonstrated that the root growth of tomato seedlings treated with DADS was strongly correlated with the alteration of the mitotic index and the number of cells in the meristematic zone. However, the effect of DADS on the mature cell length in tomato roots still requires investigation.

The root is a region of active cell division and elongation and is also a region in which important phytohormones are synthesized (Müller et al., 1998). Auxins are involved in regulating cell division and elongation (Cleland, 1995). In our results, the content of IAA in tomato roots increased as the DADS concentration increased (Figure 5). Exogenous auxins are thought to induce rapid elongation in plant tissues by increasing the mechanical extensibility of the cell wall (Taiz, 1984; Cosgrove, 1993). A small increase in IAA content can promote root growth. However, excess IAA inhibits growth. It has been reported that the dose-response curves for IAA-induced growth by elongation were bell-shaped with an optimal concentration at 10^−5^ M IAA in maize (*Zea mays* L.) coleoptile segments (Polak et al., 2011). The kinetics of IAA-induced growth rate responses are dependent on the auxin content and the plant species (Karcz et al., 1999; Martínez et al., 2011). An increase in the root auxin content promotes the biosynthesis of the ethylene precursor 1-aminocyclopropane-1-carboxylic acid (ACC) in the root and the diffusion and accumulation of ethylene, which has the ability to inhibit growth in the root elongation zone. Enhanced concentrations of auxins and ethylene emission in the elongation zone inhibit cell growth (Soltys et al., 2012). We can infer that the increase in IAA content caused by low levels of DADS is within the optimal concentration for tomato and therefore promoted root length elongation, whereas the excess of IAA caused by high concentrations of DADS increased the release of ethylene and eventually inhibited root growth.

Auxin function depends on its synthesis and distribution in plants. The flavin monooxygenases (FMOs) encoded by *YUCCA* genes catalyze the rate-limiting step in plant IAA biosynthesis (Expósito-Ródriguez et al., 2007). The transgenic potato (*Solanum tuberosum* L.) and woodland strawberry (*Fragaria vesca* L.) overexpressing *AtYUC6* and *FvYUC6*, respectively, showed developmental phenotypes associated with high-auxin content, which means that *AtYUC6* and *FvYUC6* positively regulate IAA synthesis (Kim et al., 2013; Liu H. et al., 2014). The *Arabidopsis* activation-tagged mutant *yuc7-1D* also exhibited auxin-overproduction phenotypes (Lee et al., 2012). *FZYs*, the genes encoding tomato YUCCA-like FMOs, have been reported in previous studies (Expósito-Ródriguez et al., 2007, 2011). In this experiment, the expression levels of *FZY4* and *FZY5* were increased after 0.1 mM DADA treatment, and the expression levels of all *FZYs* were promoted by the application of 6.2 mM DADS, which is consistent with the IAA content increasing in tomato root. These results suggest that the DADS-induced increases in IAA content may be caused by promotion of the expression of IAA biosynthesis genes *FZYs*. Auxin distribution and polar transport are mediated by PIN proteins. The expression of *SlPINs* varies over time and space. *SlPIN5* was mainly expressed in the seeds/locular tissue, where auxin accumulates during fruit expansion, while *SlPIN9* and *SlPIN10* were preferentially expressed in tomato root (Pattison and Catalá, 2012). In our study, upon treatment with 6.2 mM DADS, the expression levels of *SlPIN4, SlPIN9*, and *SlPIN10* were persistently increased, and that of *SlPIN5* was significantly induced. This indicates that DADS may affect the distribution of IAA by inducing the expression of *SlPIN4, SlPIN5, SlPIN9*, and *SlPIN10* in tomato root.

Cytokinins regulate cell division and engage in crosstalk with auxins as negative regulators in processes such as mitosis and root growth during root development (Werner et al., 2003; Aloni et al., 2006; Dello Ioio et al., 2008; Vanneste and Friml, 2009; Zheng et al., 2011). However, in our study, the variations in ZR content in DADS-treated tomato roots were similar to the changes observed in IAA content, and ZR reached its maximum level at the highest concentration of DADS (Figure 5), at which point significant decreases in root mitotic activity and meristem size were recorded (Table 2 and Figure 4); a small increase in ZR content after treatment with a low level of DADS (0.41 mM) led to the longest meristematic zone (Figure 4). This suggests that the highest concentration of DADS significantly increased the ZR content, inhibited root mitotic activity and reduced root meristem size, whereas the slight increase of ZR caused by a low level of DADS promoted mitotic activity. Similarly, de Vries et al. (2016) reported that cytokinin promoted root meristem development and increased meristem size in *Azolla filiculoides* Lam. However, other researchers have reported that the meristematic cells of the root meristem accumulated and that root meristem size was enlarged in *Arabidopsis* mutants deficient in cytokinin biosynthesis or signal transduction (Dello Ioio et al., 2007), which indicates that excess cytokinins inhibit mitotic activity. This may be because, in higher plants, zeatin, ZR, dihydrozeatin riboside [(diH)ZR], N^6^-(Δ^2^-isopentenyl) adenine (iP), N^6^-(Δ^2^-isopentenyl) adenosine (iPA) and kinetin (KT) are considered the major endogenous cytokinins (Lu et al., 2008); however, these substances have not been systematically examined during DADS treatment. Whether the presence of DADS could lead to a significant change in the total root cytokinin content must be investigated.

Gibberellins (GAs) play indispensable roles in seed germination, normal root development and keeping roots long and slender (Thomas et al., 2005). Artificial GA depletion can cause abnormal expansion and suppression of root elongation. GAs promote plant growth by opposing the effects of nuclear DELLA protein growth repressors, such as *Arabidopsis* RGA (repressor of *gal-3*; Fu and Harberd, 2003). The cellular responses to GAs during root growth are also positively regulated by auxin. The attenuation of auxin transport or signaling delays the GA-induced disappearance of RGA from root cell nuclei (Richards et al., 2001; Fu and Harberd, 2003). We found that the GA content of tomato roots fluctuated after DADS treatment and was highest at the lowest level of DADS. When GA and IAA reached their appropriate levels at 0.41 mM DADS, their most significant growth promoting effect was observed. This finding may indicate that IAA and GA coordinately regulate tomato root growth after DADS treatment.

Root growth arises from the proliferation of meristematic cells, followed by cell expansion that results in root elongation. Expansins, a group of plant cell wall-loosening proteins that confer extensibility to plant cells by modifying the cross-links between cellulose microfibrils and polysaccharides, are thought to be key regulators of cell wall extension during plant growth (Sharova, 2007). The biological roles of expansin genes are diverse but can be related to the action of expansins in loosening cell walls, such as during cell enlargement and root hair growth (Cosgrove et al., 2002). In the present study, increased expression of *EXPB2* and *beta-expansin precursor* was observed after 3 d, and increased expression of *EXP1, EXPB3*, and *EXLB1* was observed after 5 d, which was consistent with the finding that the RRE of tomato increased gradually after 1, 3, and 5 d of DADS treatment (Figure 7). Several studies have revealed that *EXP1* is abundant in ripening fruit and have proposed that it contributes to cell wall disassembly by non-hydrolytic activity, possibly by increasing the accessibility of the substrate to other enzymes (Powell et al., 2003; Kaur et al., 2010). The expansin precursor gene *EXPB2* is involved in cell wall modification during the early development of tomato fruit (Bemer et al., 2012). This indicates that the observed increase in the RRE value may result from the upregulation of multiple expansin genes occurring at different times after DADS treatment. However, the increase in cell size may instead be a result of compensation by these expansin genes. Expansin genes are also involved in growth processes regulated by GAs and IAA. Ding et al. (2008) found that IAA induced the expression of *EXPA1* and *EXPB3* in rice (*Oryza sativa*). *EXP1* was also induced by GAs in creeping bentgrass (*Agrostis stolonifera* L.) and gladiolus (*Gladiolus grandiflorus*) (Azeez et al., 2010; Zhou P. et al., 2011). These results imply that DADS increases the contents of IAA and GAs in tomato roots and then leads to the induction of certain expansin genes, finally resulting in the promotion of cell wall extension and root elongation.

Tomato *EXP9* plays a role in the cell expansion that accompanies cell division in the upper stem but is expressed at lower levels in tomato roots (Reinhardt et al., 1998). In the submeristematic zone of the tomato shoot apex, *EXP9* mediates cell expansion when cell division stops (Vogler et al., 2003). Thus, the decreased expression of *EXP9* (Figure 7) may explain the increases in the mitotic index and meristem size in tomato roots after DADS treatment.

In the present study, we observed that the expression of *EXP18* was downregulated after DADS treatment for 1 and 5 d (Figure 7). Caderas et al. (2000) found that tomato *EXP18* expression did not correlate with elongation, but in the meristem proper of the shoot, the gibberellin-independent *EXP18* mediates the cell expansion that accompanies cell division (Vogler et al., 2003). Thus, there may not be any direct relationship between the downregulation of *EXP18* expression and the increases in RRE and GA content after DADS treatment. However, Soltys et al. (2012) found that improper cell remodeling and the inhibitory effect in tomato roots may be caused by the downregulation of two expansin genes, *EXP9* and *EXP18*. Taken together, these data suggest that the regulation of root growth is controlled, at least in part, by the differential regulation of multiple expansin genes at different developmental stages.

## Conclusion

All data presented suggest that the allelopathic effect of DADS on tomato root growth is a complex phenomenon. DADS promoted tomato root growth at low concentrations (0.01–0.62 mM) but inhibited root growth at high concentrations (6.20–20.67 mM). These results highlight the mode of action of DADS as an allelochemical that has a biphasic, dose-dependent effect on tomato root growth, probably through increasing the content of phytohormones (IAA, ZR, and GA). Finally, these phytohormones affect mitotic activity and cell length in the root meristem and cell wall remodeling by changing the expression of expansin genes.

## Author contributions

All authors contributed to the experiment and manuscript. FC and ZC conceived and designed the research; FC and XT performed experiments; FC and HM analyzed the data and wrote the manuscript. ZC conceptualized the research and contributed to the manuscript writing. All authors read and approved the manuscript.

### Conflict of interest statement

The authors declare that the research was conducted in the absence of any commercial or financial relationships that could be construed as a potential conflict of interest.

## Abbreviations

DADS: diallyl disulfide
IAA: indole-3-acetic acid
GA: gibberellic acid
ZR: zeatin riboside
RRE: relative root elongation
ELISA: enzyme-linked immunosorbent assay
qPCR: real-time fluorescent quantitative PCR
SE: standard error
LSD: least significant difference
*T*-test: Student's *t*-test
MIC: minimum inhibitory concentration
RGA: repressor of *gal-3*.
