# Supplementary material for: The Garlic Allelochemical Diallyl Disulfide Affects Tomato Root Growth by Influencing Cell Division, Phytohormone Balance and Expansin Gene Expression
Source: Front Plant Sci. 2016 Aug 9;7:1199. doi: 10.3389/fpls.2016.01199 (PMC4977361; doi:10.3389/fpls.2016.01199)
Supplement: Supplementary file 1 [file Table1.DOC]

**Supplementary table.** The primers of *FZYs, PINs, expasin* genes and *actin* for qPCR

| Accession | Gene | Forward primer | Reward primer |
| --- | --- | --- | --- |
| NM_001247414.1 | *FZY* | AATGCCACCGGAAAAACACC | GGCATCCCTTGCTCTGTGAA |
| HG975521.1 | *FZY3* | TGGCAACAGGATATTGCAGC | TGGAAAGCTCAATCTTGTTTCTGA |
| NM_001320500.1 | *FZY4* | TGGCAACAGGGGAAAATGCT | GAACCGAGCTACGACAAACC |
| NM_001321028.1 | *FZY5* | TGAAACTTGCGGAGTGTGGA | GAACCGAGCTACGACAGACA |
| NM_001320864.1 | *FZY6* | TGGTGTGAAAAGGCCAGAAGA | CTCCCTGAAGCCACTTGTGA |
| NM_001247234.1 | *SlPIN1* | CCTGGAAAAGTTGAGGGGCA | CCAAGTGAGGCCAAACAAGC |
| NM_001247241.1 | *SlPIN2* | AAGCACGCGATTAGCAAAGG | CTAGCCGGAGGCATTTGTGA |
| NM_001247255.1 | *SlPIN4* | AAAGCCATTCCGCAAATAGGG | CATAACACTAGCCGGAGGCA |
| NM_001247263.1 | *SlPIN5* | CGTAGCTAGCAGGTGGCATT | AAGTGCCATTGTTGCTGGAC |
| NM_001247291.1 | *SlPIN9* | AGGGGAGGAGGTGACTATGG | GGCCAATGAGGCTGGAGTAA |
| NM_001247301.1 | *SlPIN10* | TCAAAGGCAGGAAGTGGCAT | GGGGTAATGCTGCCTGAAGT |
| Solyc06g051800.2 | *EXP1* | TTGGCCAAACTTTCATCGGAA | CTGCGGGCTGCATGATTTTG |
| Solyc06g049050.2 | *EXP2* | CCCACTCCCACTCATCTCTTATC | CCCCCTCCATAGAAAGTGGC |
| Solyc06g005560.2 | *EXP9* | CCGTTTTGGTTGGTCAGTCG | GCCGCTTCAGCTCTTCTACA |
| Solyc05g007830.2 | *EXP12* | TCACTCTCCAAAGAAACATGGCT | AGAACCACAGCTCAACCCATT |
| Solyc06g076220.2 | *EXP18* | GTGGCCTTCTGTGTTGCTTC | CACGCTCCACCCATAGTACC |
| Solyc03g093390.2 | *EXPB2* | GCTGGTAGTGACGGAGGATCT | CTCCCTTGCCTGAACACGAT |
| Solyc07g054170.2 | *EXPB3* | ATCCTCTACCGCCGAACTCT | GCCCTCCAATTATGGCCCAA |
| Solyc01g090810.2 | *beta-expansin precursor* | TGGACCAACCACCATTCTCG | AGTTGCACTCCACTCGTTTGTA |
| Solyc08g077910.2 | *EXLB1* | ATGATGGTCTCGTTACCGCC | CCCTCACCACTGTCAGTCAC |
| SGN-U213132 | *Actin-2/7* | GGACTCTGGTGATGGTGTTAG | CCGTTCAGCAGTAGTGGTG |
